# The work relative value estimation assessment in China: an empirical research for common surgical procedures

**DOI:** 10.3389/fpubh.2024.1385616

**Published:** 2024-06-04

**Authors:** Jin Hao, Han Yao, Ling Kong, Yuanli Liu

**Affiliations:** ^1^School of Health Policy and Management, Chinese Academy of Medical Sciences and Peking Union Medical College, Beijing, China; ^2^Department of Operation Management, Beijing Hospital of Traditional Chinese Medicine, Capital Medical University, Beijing, China; ^3^Central Laboratory, Key Laboratory for Neurodegenerative Disease of Ministry of Education, Beijing Institute for Brain Disorders, National Clinical Research Center for Geriatric Disorders, Xuanwu Hospital Capital Medical University, Beijing, China

**Keywords:** relative value scales, surgical procedures, pricing, empirical research, China

## Abstract

**Objectives:**

China’s National Health Service Items Standard (NHSIS) establishes a relative value system and plays an important role in pricing. However, there are few empirical evaluations of the objectivity of the NHSIS-estimated relative value.

**Methods:**

This paper presents a comparison between physician work relative value units (wRVUs) estimates for 70 common surgical procedures from NHSIS and those from the U.S. Medicare Physician Fee Schedule (MPFS). We defined the ratio of the wRVUs for sample procedures to the benchmark procedure (inguinal hernia repair) as a standardized relative value unit (SRVU), which was used to standardize the data for both schedules. We examined the variances in the ranking and quantification of SRVUs across specialties and procedures, as well as how SRVUs impact procedure reimbursement prices between the two schedules.

**Results:**

There was no systematic difference between MHSIS-estimated SRVUs and MPFS-estimated, but the dispersion of MPFS-estimated SRVU was greater than that of MHSIS-estimated, and the discrepancies increased with surgical risk and technical complexity. The discrepancies of SRVUs were significant in cardiothoracic procedures. Additionally, whether SRVUs were based on MPFS or MHSIS, there was a positive association between them and payment prices. However, in terms of the impact of SRVUs on payment pricing, the NHSIS system was lower than the MPFS system.

**Conclusion:**

China has made incremental progress in estimating the relative value of healthcare services, but there are shortcomings in valuation methods and their impact on pricing. The modular assessment method should be considered as a component to optimize reform.

## 1 Introduction

Globally, diverse nations have implemented medical service pricing policies as a pivotal policy tool to manage medical expenditure growth, optimize resource allocation, and promote value-based payment (1–3). The objective is to establish scientific and rational prices for medical services, thereby stimulating the motivation of medical providers and guaranteeing that patients receive excellent, efficient, and economical healthcare services (4). Consequently, determining the optimal balance between physicians’ desire for increased remuneration and the affordability constraints of patients and medical insurance funds poses a universal challenge worldwide.

China, similar to other countries, emphasizes the creation of a medical service pricing system that truly embodies the value of technical labor, serving as a cornerstone of its healthcare pricing reform. To standardize pricing units, China has established the Chinese Classification of Health Interventions (CCHI), a comprehensive terminology system encompassing medical service coding, standard names and operational definitions. Additionally, in promoting of healthcare pricing reform, China has been drawing inspiration from the Resource-Based Relative Value Scale (RBRVS) since 2012. This involves exploring a relative technical labor value system tailored for Chinese medical procedures, considering factors like working hours, technical complexity, and risk level.

The RBRVS was developed in the late 1980s by Harvard researchers led by William Hsiao, utilized national surveys and statistical analyses anchored in the Current Procedural Terminology (CPT) framework (5). In 1989, the U.S. Physician Payment Review Commission integrated the RBRVS into a proposed Medicare fee schedule presented to Congress. Congress then passed legislation that year, establishing the Medicare Physician Fee Schedule (MPFS), implemented in 1992. This schedule based payment pricing on the relative resource consumption of services, rather than other factors, such as historical charges (6). Following this, nations like Canada (7), Japan (8), and South Korea (9) adopted the RBRVS methodology to create their own localized, cost-based relative value systems for medical services. In 2012, China introduced the resource-based relative value as a critical component in determining medical service pricing within its National Health Service Items Standard (NHSIS) (10). The October 2023 NHSIS update further refined this system, positioning it as a precise scientific tool for evaluating resource consumption, pricing, and clarifying the price comparison relationship among medical procedures (11).

The resource consumption in medical service procedures typically encompasses technical labor costs, medical supplies and facilities costs, as well as other administrative costs (such as professional liability insurance premiums covered by the Medical Insurance System). Technical labor costs - referred to as the work relative value units (wRVUs) in the RBRVS model - represent the most complex component of cost measurement and are a focal point for localized improvements under the RBRVS model across various countries. Especially for surgical procedures, which account for a high proportion of medical services, involve various operation methods, and cover multiple departments, the estimation of wRVUs and pricing are particularly complex (12). International experience (13, 14) demonstrates that discussions on the rationality of evaluation methods and results for the relative value of surgical procedures accompany virtually the entire process of system establishment and improvement. Therefore, during this critical period of establishing relative value system and reforming pricing policies in China, it is necessary to conduct a scientific evaluation of the system. Nevertheless, empirical research in this area remains scarce.

Given the complexity and manual nature of medical work, it is difficult to establish a “gold standard” to judge the final wRVUs. Consequently, this study attempts to investigate a methodology facilitating cross-regional comparisons of relative value, leveraging the MPFS founded on RBRVS as a frame of reference. Through a comprehensive evaluation of relative value assessment methods in both China and the United States, we introduce a standardized wRVUs model that is suitable for cross-regional comparison. Utilizing this framework, we selected several common surgical procedures as case studies to perform an international comparative analysis, focusing on the wRUVs valuation outcomes and their influence on pricing. This research not only bridges the gap in empirical studies regarding China’s relative value system assessment but also provides methodological insights for nations that established their own relative value systems by drawing on RBRVS to carry out related research.

## 2 Methods

### 2.1 wRVUs valuation methods of MPFS and NHSIS

**Figure 1:**
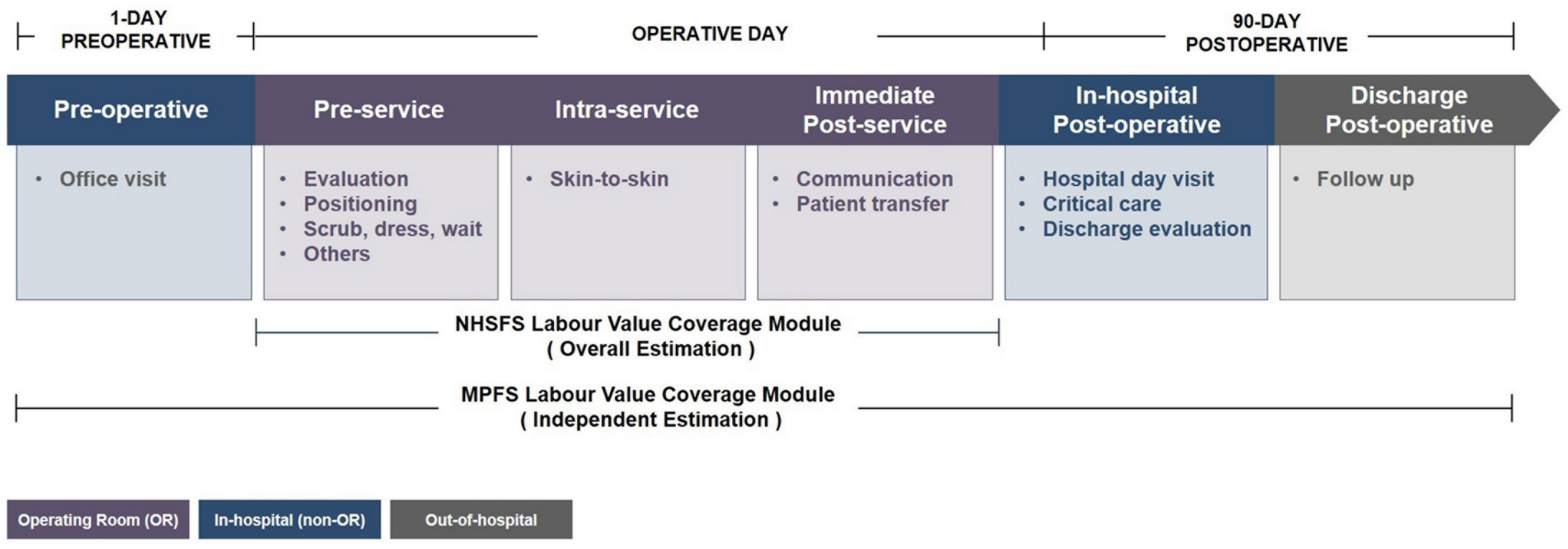
The work relative value assessment modules of surgical procedures for NHSIS and MPFS. The figure shows the flow modules of the surgical procedure in the global period from 1-day preoperative to 90-day postoperative, and the main services of each module. The purple module indicates physician activity within the operating room, the blue module indicates activity within the hospital other than the operating room, and the gray module indicates activity outside the hospital. MPFS, The U.S. Medicare Physician Fee Schedule; NHSIS, National Health Service Items Standard.

Researchers in China have restructured the wRVU valuation components from the RBRVS study to estimate the wRVUs of the NHSIS, employing the same cross-specialty alignment statistical techniques as the Harvard University research team and leveraging a knowledge-intensive expert consultation approach. The consistency of the theoretical basis between the NHSIS wRVU estimates and MPFS (see Supplementary Table S1) establishes a solid foundation for comparative research. Specifically, the total wRVUs valuation of a surgical procedure can be considered the sum of pre-, intra-, and post-service work. As depicted in Figure 1, the MPFS additionally incorporates evaluation and management codes into the total wRVUs for procedures with a 10- or 90-day global cycle, thereby encouraging clinicians to oversee perioperative and postoperative care (15). This distinction underscores the key difference in the estimation and pricing of surgical wRVUs between China and the United States. However, given that MPFS utilizes a modular valuation approach, individually assessing each component’s relative value and providing comprehensive data for each module, we can use these data to adjust the relative value of Chinese and American surgical programs to the same coverage through mathematical changes.

### 2.2 Sources of data

We obtained data from three publicly available sources: (1) NHSIS Version 2023 (11), (2) MPFS of the calendar year 2023 (16), and (3) the health care service procedures fees schedule of Beijing (17). Because all data were publicly available and no patient identifiers were included, this analysis did not meet the definition of human subjects research, and institutional review board and informed consent were waived.

We obtained the relative technical difficulty coefficient (0–100), relative potential risk coefficient (0–100), and total work RVUs of surgical procedures from NHSIS files, which were matched to the payment price from the fees schedule of Beijing by codes. The MPFS files provided the work RVUs and the percentage of the intraoperative portion of the global package that could be used to calculate the intraoperative work RVUs for each procedure. The conversion factor and practice expenses RVUs used to calculate the payment price were also obtained from these files. Furthermore, we accurately matched procedures by CCHI and CPT description and then integrated Chinese and American data by codes (see Supplementary Table S2).

### 2.3 Study sample

**Figure 2:**
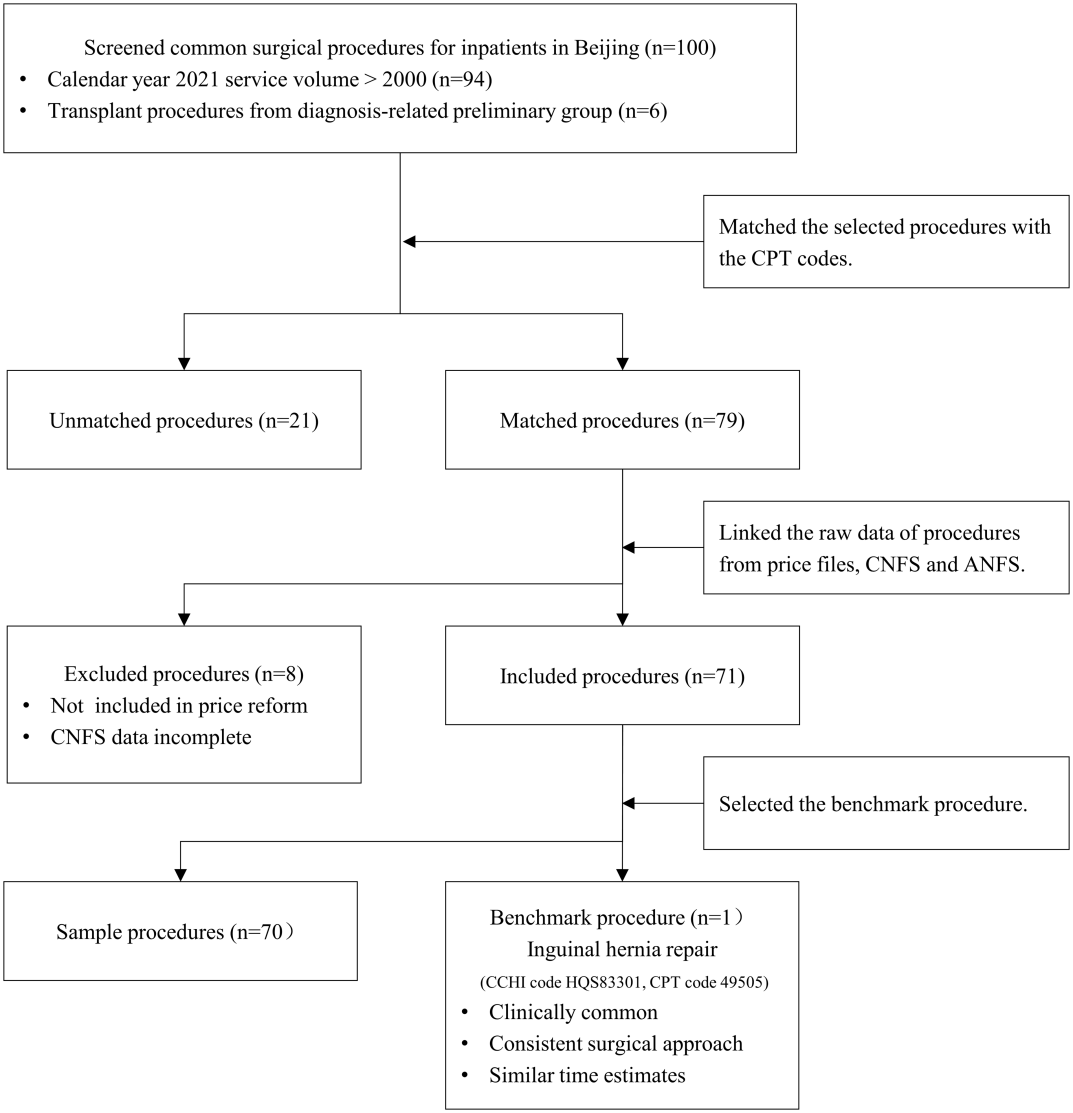
Flow chart of sample selection. MPFS, The U.S. Medicare Physician Fee Schedule; NHSIS, National Health Service Items Standard.

An advisory panel consisting of 10 experienced surgeons from various subspecialties oversaw the selection of sample and benchmark procedures. Figure 2 illustrates the screening flow for the sample procedures. Using the medical records of inpatient surgical patients in Beijing from 2021 as a basis, we initially filtered out procedures with an annual service volume exceeding 2,000 (encompassing 94 procedures) and all transplant procedures from the preliminary group of diagnosis-related groups (consisting of 8 procedures). These datasets collectively made up the candidate pool. In a secondary screening process, the advisory group excluded two transplant procedures with unclear surgical site descriptions - “living donor related transplant” and “living donor unrelated transplant” - based on volume, clinical relevance, and representativeness within their respective specialties. This refinement left us with 100 common surgical procedures. Following this, the advisory panel employed the standardized terminology sets for surgical operations used in China and U.S, namely CCHI and CPT, as a tool to precisely match selected common surgical procedures with the MPFS, based on the criterion of consistency in the method of operation. Ultimately, 79 procedures were successfully matched. Subsequently, we integrated raw data for each procedure from various sources, including price documents, NHSIS, and MPFS. However, 5 procedures were excluded as they had not been included in the price reform, and 3 procedures lacked complete NHSIS data. Therefore, our final study sample encompassed 71 procedures, accounting for approximately 61% of all documented surgical procedures in Beijing inpatient medical records for 2021.

### 2.4 Estimation of standardized relative value unit

Given the disparities in regional economies and healthcare delivery systems, a direct comparison of labor value or payment rates for surgical procedures between China and the United States may not be appropriate. Therefore, our study established a standardized relative value unit (SRVU) by defining the quotient of the total work RVU for sample procedures relative to a benchmark procedure. The advisory panel selected inguinal hernia repair (CCHI code HQS83301, CPT code 49505) as the benchmark procedure, based on its clinical commonality, consistent surgical approach, and comparable time estimates. To estimate MPFS-estimated relative labor value, we scaled each procedure’s wRVU by the proportion of intraoperative time (OPi). The detailed computational modeling was presented in Formulas (1) and (2). The same method was used to calculate the standardized payment price unit (SPPU) when comparing payment prices.

(1) $$SRVU_{\mathrm{NHSIS},i}=\frac{wRVU_{i}}{wRUV_{\mathrm{benchmark}}}$$

(2) $$SRVU_{\mathrm{MPFS},i}=\frac{wRUV_{i}\times OP_{i}}{wRUV_{\mathrm{benchmark}}\times OP_{\mathrm{benchmark}}}$$

### 2.5 Statistical analysis

We compared the difference in the distribution of SRVUs for surgical procedures in NHSIS and MPFS by plotting a waterfall plot and dumbbell diagram. A comparison of SRVUs for procedures between NHSIS and MPFS using the paired-sample Wilcoxon signed-ranks test. To comprehend how discrepancies in the overall data were manifested throughout surgical specialties, we categorized sample procedures into 6 specialties and conducted subgroup analyses. In addition, linear regression analysis was used to assess the relationship between SRVUs and payment prices for both the NHSIS and MPFS data. R-squares were then determined for each dataset to compare the magnitude of the effect of SRVUs on payment prices between the two fee schedules. All analyses were performed using R for Windows (version 4.3.1), with statistical significance determined using a 2-tailed alpha risk of 0.05 or less.

## 3 Results

### 3.1 Overall discrepancies in SRVUs

**Figure 3:**
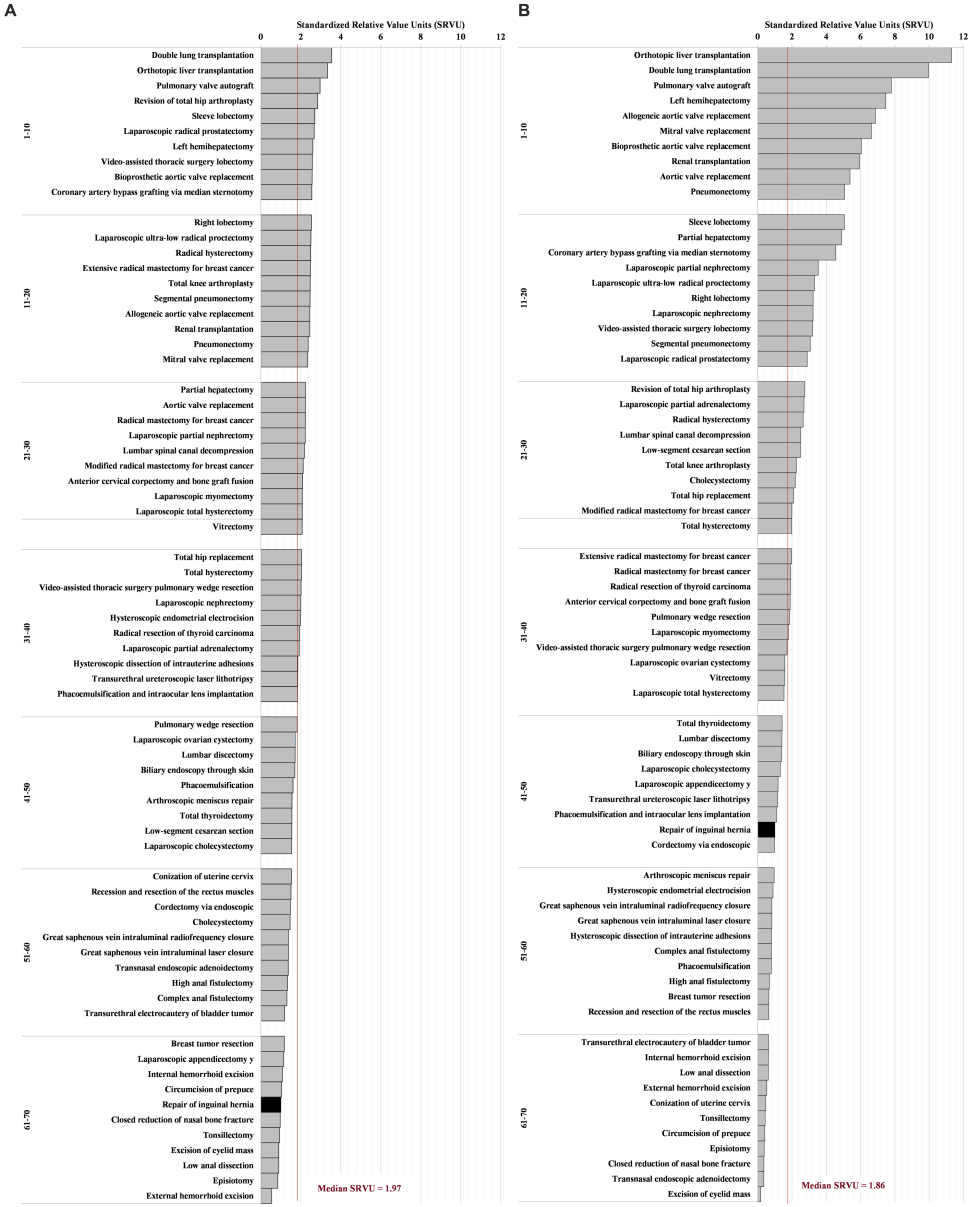
The SRVUs of 70 Common Surgical Procedures for NHSIS and MPFS. **(A)** Is the distribution of NHSIS-estimated standardized work relative value of surgical procedures. **(B)** Is the distribution of MPFS estimates for the same procedures. Each bar represents a procedure. The black bar indicates the benchmark procedure. The red horizontal line representing the overall median. The procedures were ranked in descending order based on the SRVUs, and then divided into intervals of each 10 rankings. MPFS, The U.S. Medicare Physician Fee Schedule; NHSIS, National Health Service Items Standard; SRVUs, Standardized work relative value units.

**Figure 4:**
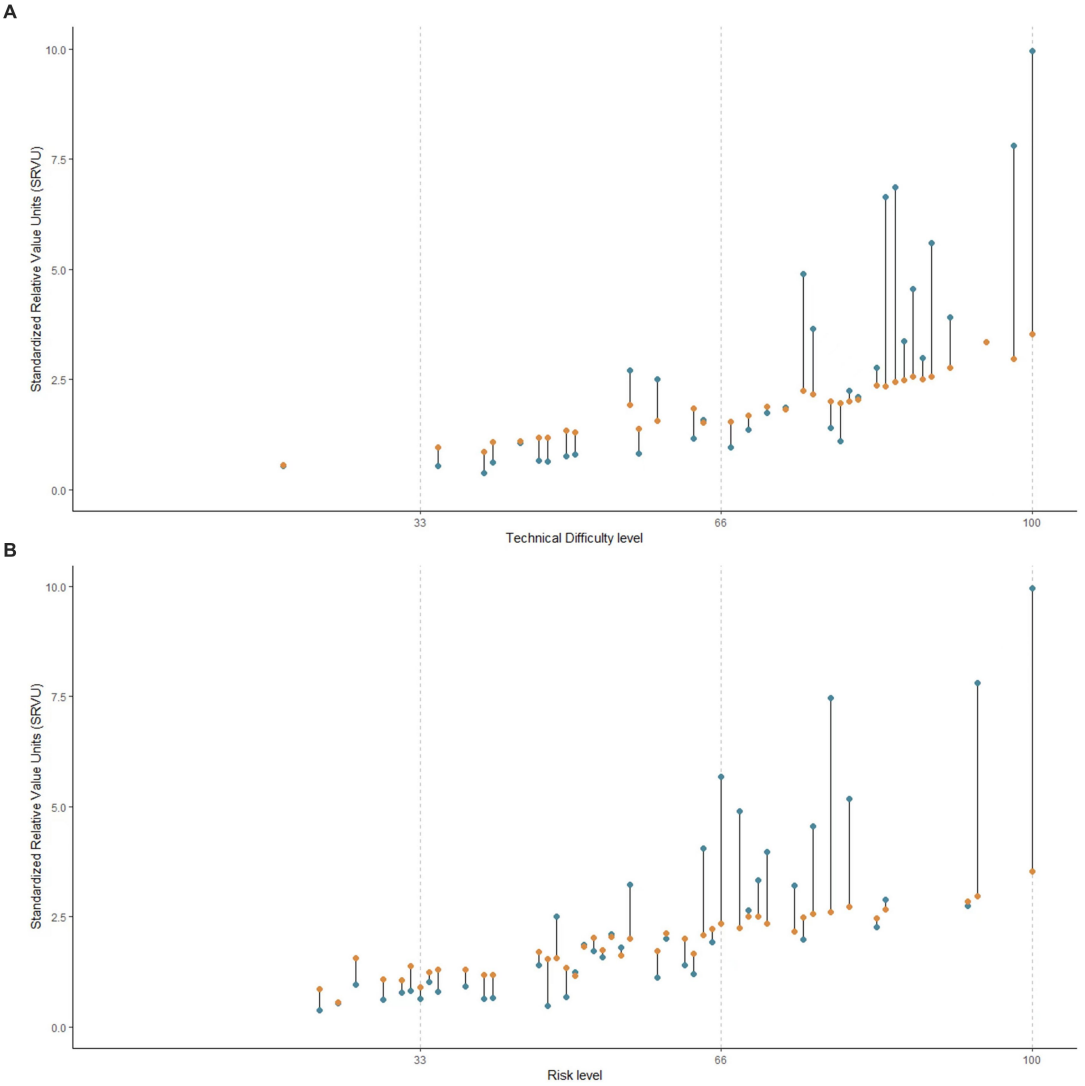
The Association between the Discrepancies of SRVUs and Procedures’ Difficulty or Risk Level. **(A)** Is the association between the discrepancies of SRVUs and NHSIS-based technical difficulty level of procedures. **(B)** Is the association between the discrepancies of SRVUs and NHSIS-based risk level of procedures. The NHSIS estimated the relative technical difficulty and potential risk of surgical procedures using integers ranging from 0 to 100 and divided them into three intervals: low, medium, and high, as shown by the dashed line in the figure. The orange circle represents the median NHSIS-estimated SRVUs of procedures corresponding to the technical difficulty or risk level on the horizontal axis, and the blue circle represents the median MPFS-estimated SRVUs of the same procedures. MPFS, The U.S. Medicare Physician Fee Schedule; NHSIS, National Health Service Items Standard; SRVUs, Standardized work relative value units.

The estimated SRVUs for 70 sampled procedures from both the NHSIS and MPFS datasets are presented in Figures 3A,B. The median values estimated by MHSIS and MPFS were 1.97 and 1.86, respectively, demonstrating a non-significant statistical difference (*p* = 0.401). Our research revealed that the dispersion of SRVUs in MPFS (with a range from 0.17 to 11.31) exceeded that in NHSIS (with a range from 0.54 to 3.53). This has led to operations with similar rankings could have vastly different SRVUs. Furthermore, these discrepancies positively correlated with both the relative technical difficulty (regression coefficient = 0.44, *p* < 0.001) and the relative risk (regression coefficient = 0.46, *p* < 0.001) of the procedures, as assessed by NHSIS. This suggests that as surgical risk or technical complexity increases, so does the divergence in SRVU estimation between NHSIS and MPFS. (refer to Figures 4A,B).

### 3.2 Discrepancies in SRVUs across specialties

**Figure 5:**
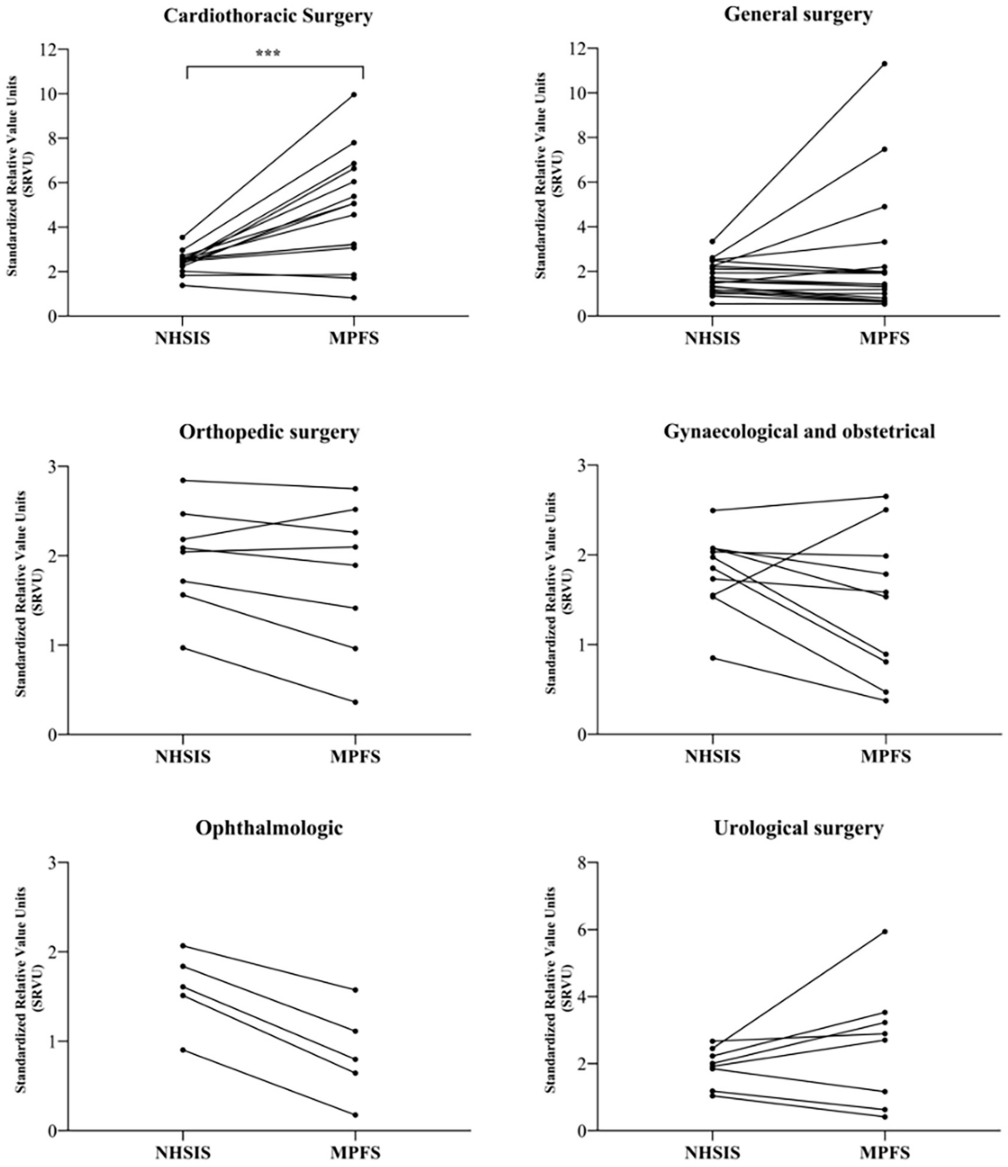
The SRVUs across 6 Surgical Specialties for NHSIS and MPFS. The figure shows the trend of paired changes in the same surgical procedures for six specialties. ***p < 0.01. MPFS, The U.S. Medicare Physician Fee Schedule; NHSIS, National Health Service Items Standard; SRVUs, Standardized work relative value units.

Figure 5 shows the SRVUs of surgical procedures across specialties, comparing MPFS and NHSIS estimations. The NHSIS-estimated SRVUs for each specialty are arranged in descending order and connected to their corresponding MPFS estimations using line segments. Intersections of these lines indicate differing relative rankings for procedure SRVUs between NHSIS and MPFS. Our research found that specialty rankings primarily shifted between adjacent positions, with minimal changes in magnitude. (refer to Supplementary Figure S1) The slope of each line segment reflects the extent of the difference in SRVU readings, highlighting significant disparities in cardiothoracic (*p* = 0.004), with no systematic difference in the SRVUs of the other five specialties.

### 3.3 Discrepancies in the effect of SRVUs on pricing

**Figure 6:**
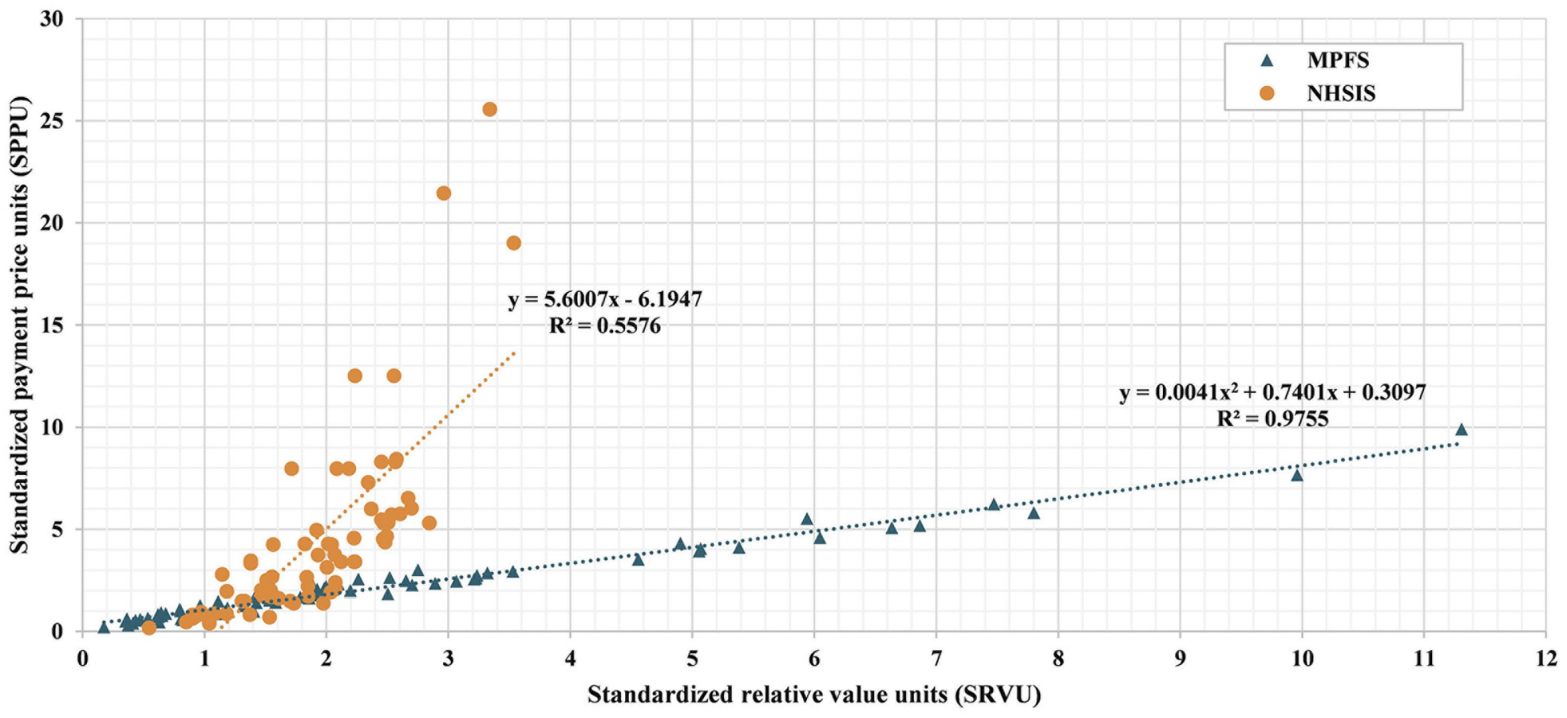
Scatter plot of wRUV estimates and payment prices for 70 Common Surgical Procedures. The figure shows the distribution for each of the 70 procedures in a coordinate series where the standardized work relative value unit (SRVU) is the x-axis and the standardized payment price unit (SPPU) is the y-axis. Orange circles and blue triangles, respectively, depict the calculation results of NHSIS and MPFS. The NHSIS linear correlation trend is plotted as an orange dashed line, while the MPFS linear correlation trend is plotted as a blue dashed line. The regression equations and R-squared values are labeled next to each line. MPFS, The U.S. Medicare Physician Fee Schedule; NHSIS, National Health Service Items Standard.

The link between relative values and the total payment price for intraoperative work is illustrated in Figure 6. Scatter plots demonstrate a positive correlation between SRVUs and standardized payment price units (SPPUs), regardless of the dataset used (NHSIS or MPFS). Linear regression analyses demonstrated that SRVUs accounted for 55.76% of the variation in payment prices based on NHSIS data, whereas they explained 97.55% of the variation when using MPFS data.

## 4 Discussion

This study proposed a standardized analytical framework for cross-regional comparisons of the relative value of medical services. By comparing the relative value assessment methods and valuation results of surgical procedures between China and the United States, the study evaluated the present state of the Chinese system and put forth recommendations for optimization.

### 4.1 Sino-US comparison of surgical relative value and pricing: similarities, differences, and drivers

This study revealed that there are no overall significant discrepancies in SRVUs for common surgical procedures between China and the United States, indicating that China’s evaluation of the relative labor values for surgical procedures aligns well with the international consensus. Nonetheless, considering that the overall compensation for medical services hinges on both price and service volume, variations in the valuation of individual procedures may be amplified or minimized at different levels - physician, specialty, and hospital - owing to diverse service volumes, thereby influencing pricing or compensation equity (18). Indeed, our subgroup analysis uncovered such disparities at the specialty level (refer to Figure 5). Consequently, it becomes imperative to investigate the impact of these relative value differences on payment and compensation outcomes within the context of specific service volumes in subsequent research.

Our findings indicate that the dispersion in the relative labor value in China is narrower compared to the United States. Additionally, the valuation disparity between the two countries exhibits a positive correlation with the level of technical difficulty and risk. This suggested a potential distortion in the current assessment of the relative labor value for high-tech surgical procedures in China. Notably, challenging, high-risk, and labor-intensive procedures are the main breakthroughs in the ongoing dynamic pricing adjustments for medical services in China. Therefore, an accurate assessment of the relative value of such procedures is crucial. Our results underscore the necessity of prioritizing this type of surgical procedure in future optimizations of the relative value system for medical services.

Further examination of the correlation and strength of the relationship between relative value and reformed payment prices revealed that, in comparison to the United States, the contribution of physician labor value to total surgical payment price is smaller in China (refer to Figure 4). These results can be understood in light of the characteristics of the Chinese medical price policies (19–21), which include the following: (1) Fees for professional work tend to be low in comparison to fees for materials and equipment. (2) The NHSIS has packaged some medical supplies with surgical procedures to control the sensible use of consumables in medical facilities. As a result, some procedure payment prices include expensive material costs. (3) In China, the procedure price is mainly used for payments to medical institutions, whereas in the United States, the MPFS is used for physician fee payments. The significant proportion of medical supplies and overhead costs likely explains the lower contribution of physician labor value to surgical prices in China. Additionally, these disparities are also associated with distinct relative value systems in the U.S. and China, as well as variations in how these systems connect to pricing.

### 4.2 Optimizing relative value and pricing system in China: approaches and challenges

China’s relative value system, based on the RBRVS, shares high consistency with the United States in terms of theoretical foundation and survey methods. However, there are still some differences in details regarding relative value assessment and value-based pricing mechanisms. In the United States, a modular approach is adopted for relative value assessment (22). Resource consumption assessment methods are developed separately based on the characteristics of technical labor costs, medical consumables, equipment and facilities costs, and other management costs to improve the accuracy of valuation. The sum of all parts represents the total relative value of the procedure. Based on this, the MPFS enables direct conversion between relative value and payment price through a nationally unified price factor. Different regions can set geographic practice cost indices to correlate with the regional economy. This approach clearly reflects the proportion of each cost factor in pricing. In contrast, China’s current relative value system has not yet achieved the correlation of relative values for different cost elements, and the linkage between relative value and price is also ambiguous. This lack of transparency in pricing may affect the correspondence between price and value, especially the value of technical labor.

Despite distinct medical service pricing and payment systems in the U.S. and China, both share a common objective: purchasing services based on value. Consequently, the modular relative value valuation method employed by the MPFS offers a valuable reference for assessing relative values and facilitating dynamic price adjustments for medical service projects in China. Firstly, clearly compensating technical labor activities facilitates the communication of incentive orientation. Additionally, this transparency encourages professional societies to actively assess the value of their activities and collaborate with policymakers to establish rational pricing. Secondly, with national centralized procurement, the costs of medical supplies are expected to fluctuate significantly in the future. Thus, independent pricing of medical supplies would facilitate dynamic price adjustments. The Pilot Program for Deepening the Reform of Medical Service Prices in China has identified the separation of technology and consumption as a key reform objective, aiming to gradually decouple medical supplies from procedures payment price. Thirdly, pertinent research demonstrated that institutions with different levels may result in variations in practice consumption (23, 24), and the inputs of physician activity may vary depending on patient differences (12, 25). Modular evaluating mechanisms provide a solid foundation for differentiated regulation, with adjustment factors for the technical labor activities module potentially including risks, complexity, and HTA results. The level of medical institutions can inform adjustments to the practice cost module. This modular valuation optimization suggestions based on our findings are also applicable to non-surgical procedures, as this model aligns well with the overall goals of China’s medical service price reform.

Since its inception, the RBRVS has been widely acknowledged as a reasonable and equitable approach for evaluating healthcare service inputs. However, previous studies (12, 22, 26) have demonstrated that it is not without its limitations: (1) complex and drawn-out treatment procedures may be preferred over straightforward ones for specific conditions; (2) demand-side considerations, such as individual variations in clinical complexity and potential health benefits, are completely ignored when evaluating medical services; and (3) an insensitivity to the efficiency improvements and cost reductions brought about by technological advancements (as clinical doctors gain experience, their workload may decrease over time, but the relative value may not decrease accordingly). These shortcomings pose challenges for pricing and payment mechanisms grounded in the relative value system. Nonetheless, practices in some nations (27) have demonstrated that targeted policy interventions can effectively address imbalances in service provision and promote alignment between the relative value system and value-based payments. For instance, the Japanese government gradually reduced MRI scan prices over 6 years to address service volume surges and optimize service mix. Similarly, the Affordable Care Act in the United States increased primary care fees to ensure adequate compensation for hospitals providing or facilitating access to services. China’s ongoing price reform is also exploring avenues to guide doctors to make medical decisions based on patients’ needs. For example, this study revealed that, despite endoscopic surgery often requiring a higher resource consumption compared to traditional open surgery for similar procedures, the government has established an identical payment price for both in its price reform efforts.

### 4.3 Study limitations and future prospects

When interpreting our research results, several limitations must be acknowledged. Firstly, our study utilized a limited sample size. Previous research suggests that the majority of surgical services stem from a small subset of procedures (28). In our study, we carefully selected 70 prevalent surgical procedures encompassing 10 organ systems and 6 specialties, guided by surgeon consensus and the inpatient surgical service volume. Nonetheless, extrapolating these findings necessitates consideration of the sample’s representativeness. Secondly, our study was an international comparison of relative values based on benchmark procedures. On one hand, the standardized results of this study are inevitably influenced by the selection of benchmark procedures. On the other hand, our research prefers to indirectly suggest improvements by identifying significant differences between China’s relative value system and pricing outcomes compared to internationally mature systems, rather than directly giving advice on increasing underestimated values or decreasing overestimated values. Thirdly, it should be noted that our study was conducted within a pilot area. In China, healthcare pricing reform allows local governments to determine the payment price, while the central government sets the service items. The pace of the reform varies from region to region. Expanding the scope of the study may introduce additional confounding factors due to the complex and uncertain regional differences in healthcare price reform. Finally, the relative value and pricing data for surgical procedures in China and the United States, obtained in this study, were specifically sourced from 2023. This provides valuable insights into evaluating the relative value system and pricing mechanisms during this period. However, it is crucial to recognize the limitations imposed by this temporal constraint. Healthcare systems and pricing mechanisms are dynamic, influenced by various factors such as medical technology advancements, health insurance policies, and the economic environment. Consequently, the conclusions drawn from data of a specific period may not be universally applicable to other timeframes. As China’s dynamic adjustment mechanism for the relative value and pricing of healthcare services evolves, future research should consider using datasets spanning longer periods. This will aid in understanding the dynamic nature of these systems and their progression over time. However, proper adjustment and standardization must be taken when handling data from different timeframes due to potential complexities arising from varying political, economic, and social factors.

## 5 Conclusion

In this U.S.-China comparison of common surgical procedures, we found that China has made incremental progress in estimating the relative value of healthcare services, but there are shortcomings in valuation methods and their impact on pricing. The modular relative value assessment method and necessary government regulatory tools should be considered as components to optimize China’s healthcare pricing reform.

## Data availability statement

The original contributions presented in the study are included in the article/Supplementary material, further inquiries can be directed to the corresponding author.

## Author contributions

JH: Conceptualization, Data curation, Formal analysis, Methodology, Project administration, Supervision, Writing – original draft, Writing – review & editing. HY: Data curation, Formal analysis, Methodology, Writing – original draft. LK: Formal analysis, Methodology, Writing – original draft. YL: Conceptualization, Funding acquisition, Methodology, Project administration, Supervision, Writing – review & editing.
