# Supplementary material for: The work relative value estimation assessment in China: an empirical research for common surgical procedures
Source: Front Public Health. 2024 Jun 4;12:1385616. doi: 10.3389/fpubh.2024.1385616 (PMC11183284; doi:10.3389/fpubh.2024.1385616)
Supplement: Supplementary file 1 [file Data_Sheet_1.pdf]

Supplementary Material

Supplementary Table 1 Comparison of key elements of work relative value assessment between China and the United States

Elements	United States	China
**Object**	**CPT** Current Procedural Terminology	**CCHI** China Classification of Health Interventions
**Developer**	**Funding Agency**<br>· Centers for Medicare & Medicaid Services (CMS)<br>· American Medical Association (AMA)<br>**Research Institution**<br>· Harvard University	**Funding Agency**<br>· National Health Commission<br>· National Administration of Traditional Chinese Medicine<br>· National Administration of Disease Control and Prevention<br>**Research Institution**<br>· China National Health Development Research Center
**Approach**	**Sub-Specialty Measure Resource-Based Work Relative Value Units**<br>· Knowledge-intensive expert consultation (100 experts participated)<br>· Nationwide physicians sampling survey (1,977 physicians participated)<br>· Statistical extrapolation to Non-Surveyed services<br>**Cross-Specialty Alignment**<br>· Selecting the linking services (the same or equivalent services in different specialties)<br>· align individual estimates onto a common scale (least squares method)	**Sub-Specialty Measure Resource-Based Work Relative Value Units**<br>· Knowledge-intensive expert consultation (＞400 experts participated))<br>**Cross-Specialty Alignment**<br>· Selecting the Linking Services (the same or equivalent services in different specialties)<br>· align individual estimates onto a common scale (least squares method)
**Expert panels**	**Technical Consulting Group**<br>· Divided into 33 specialty groups, consisting of 3-5 senior clinical experts in each specialty<br>· Defined work and its dimensions<br>· Evaluated the validity of measurement and the reasonableness of results.<br>**The Cross Specialty Panel**<br>· 34 physician subset of the technical consulting group<br>· Assisted the Harvard research group in identifying which cases appeared to be clinically the same, or clinically equivalent.	**Clinical Expert Working Group**<br>· Divided into 48 clinical professional groups, consisting of 313 clinical experts<br>· Responsible for screening, merging, sorting, and evaluating research of services.<br>**Clinical Expert Review and Demonstration Group.**<br>· Divided into 48 clinical professional groups, consisting of 195 clinical experts.<br>· Responsible for the review, demonstration, and confirmation of the results.

Supplementary Table 1 Comparison of key elements of work relative value assessment between China and the United States (continued)

Elements	United States	China
**Evaluation Components**	**Time**<br>・Pre-operative time<br>・Intra-operative time (pre-service, intra-service, and immediate post-service time)<br>・Post-operative time<br>**Mental Effort and Judgment**<br>・The amount and complexity of medical analyses, diagnostics, or operations required<br>・The urgency of medical decision-making required<br>**Technical Skill and Physical Effort**<br>・The technical skills required<br>・The physical effort required<br>**Psychologic Stress**<br>・The risk of significant complications, morbidity, and mortality<br>・The extent to which the outcome depends on the skill and judgment of the physician<br>・The estimated risk of malpractice suit with poor outcome	**Time**<br>・Intra-operative time (pre-service, intra-service, and immediate post-service time)<br>**Technical Difficulty**<br>・The amount and complexity of medical analyses, diagnostics, or operations required<br>・The technical skills required<br>**Potential Risks**<br>・The probability of complications<br>・The severity of possible complications

Supplementary Table 2 Relevant information on 70 common surgical procedures in NHSIS and MPFS

Specialties	Surgical procedure name	NHSIS-based					MPFS-based				
		Code	Work RVUs	Relative technical difficulty	Relative risk level	Payment (USD)	Code	Work RVUs	Intra-operative percentage	Practice expenses RUVs	Payment (USD)
Benchmark produce	Repair of inguinal hernia	HQS7P301	23.50	37	27	77	49505	7.96	0.81	5.78	404
Cardiothoracic Surgery	Double lung transplantation	HJE7T302	83.10	100	100	1,458	32853	84.48	0.76	29.41	3,095
	Pulmonary valve autograft	HKY6L303	69.60	98	94	1,645	33413	59.87	0.84	20.81	2,351
	Sleeve lobectomy	HJF6U303	63.40	91	80	462	32486	42.88	0.76	15.48	1,589
	Video-assisted thoracic surgery lobectomy	HJF6U501	60.50	86	80	647	32663	24.64	0.84	10.58	1,034
	Bioprosthetic aortic valve replacement	HKR6L304	60.30	89	71	637	33410	46.41	0.84	17.21	1,858
	Coronary artery bypass grafting via median sternotomy	HKU7K301	60.00	87	76	959	33510	34.98	0.84	13.59	1,421
	Right lobectomy	HJF6U302	59.50	86	80	438	32482	27.44	0.76	12.59	1,106
	Segmental pneumonectomy	HJG6U301	57.90	86	80	346	32669	23.53	0.84	10.30	994
	Allogeneic aortic valve replacement	HKR6L305	57.60	85	66	637	33406	52.68	0.84	19.13	2,095
	Pneumonectomy	HJE6W301	55.60	86	71	459	32488	42.99	0.76	16.87	1,638
	Mitral valve replacement	HKQ6L301	55.00	84	66	559	33430	50.93	0.84	19.32	2,053
	Aortic valve replacement	HKR6L302	52.50	76	64	959	33405	41.32	0.84	15.59	1,663
	Video-assisted thoracic surgery pulmonary wedge resection	HJE6U501	47.30	80	52	328	32666	14.50	0.76	7.69	619
	Pulmonary wedge resection	HJE6U302	42.80	73	51	328	32505	15.75	0.76	7.99	660
	Great saphenous vein intraluminal radiofrequency closure	HM57D302	32.40	57	32	255	36475	5.30	0.00	1.72	233
	Great saphenous vein intraluminal laser closure	HM57D301	32.40	57	32	265	36478	5.30	0.00	1.76	233
General surgery	Orthotopic liver transplantation	HQA7T302	78.50	95	80	1,961	47135	90.00	0.81	48.20	4,004
	Left hemihepatectomy	HQA6U303	61.20	89	78	441	47122	59.48	0.81	28.01	2,519
	Laparoscopic ultra-low radical proctectomy	HPU6X502	58.90	88	70	409	44204	26.42	0.81	13.59	1,157
	Extensive radical mastectomy for breast cancer	HYA6X303	58.30	83	75	335	19307	17.99	0.71	12.93	850
	Partial hepatectomy	HQA6U302	52.50	75	68	261	47120	39.01	0.81	21.17	1,745
	Radical mastectomy for breast cancer	HYA6X301	52.30	80	65	261	19305	17.46	0.71	12.74	831
	Modified radical mastectomy for breast cancer	HYA6X302	49.80	80	60	261	19306	18.13	0.71	13.91	885
	Radical resection of thyroid carcinoma	HDC6X301	45.40	80	38	286	60240	15.04	0.82	9.44	720
	Biliary endoscopy through skin	HQE6P501	40.00	69	46	114	47554	9.05	0.00	2.90	395
	Total thyroidectomy	HDC6W301	36.50	67	34	204	60220	11.19	0.82	7.92	565
	Laparoscopic cholecystectomy	HQK6W501	36.20	49	50	155	47562	10.47	0.81	6.73	503
	Cholecystectomy	HQK6W301	34.40	44	50	155	47600	17.48	0.81	10.28	808
	High anal fistulectomy	HPV6U304	31.20	44	49	113	46275	5.42	0.81	6.33	354
	Complex anal fistulectomy	HPV6U305	30.70	50	35	113	46280	6.39	0.81	6.95	401
	Breast tumor resection	HYA6U307	27.70	46	41	65	19120	5.92	0.71	5.18	310
	Laparoscopic appendicectomy y	HPR6W501	26.90	44	31	214	44970	9.45	0.81	6.32	462
	Internal hemorrhoid excision	HPV6U306	25.40	41	29	65	46255	4.96	0.81	4.80	292
	Low anal dissection	HPV6U303	21.20	35	34	61	46270	4.92	0.81	6.18	336
	External hemorrhoid excision	HPV6U307	12.80	18	24	13	46250	4.25	0.81	4.54	264

Supplementary Table 2 Relevant information on 70 common surgical procedures in NHSIS and MPFS (continued)

Specialties	Surgical procedure name	NHSIS-based					MPFS-based				
		Code	Work RVUs	Relative technical difficulty	Relative risk level	Payment (USD)	Code	Work RVUs	Intra-operative percentage	Practice expenses RUVs	Payment (USD)
Gynecological and obstetrical	Radical hysterectomy	HTD6X302	58.60	88	69	357	58200	23.10	0.74	13.32	1,005
	Laparoscopic myomectomy	HTD6U501	48.70	78	62	158	58545	15.55	0.74	8.84	673
	Laparoscopic total hysterectomy	HTD6W501	48.70	78	62	184	58570	13.36	0.74	8.64	612
	Total hysterectomy	HTD6W301	47.80	78	62	147	58150	17.31	0.74	10.19	760
	Hysteroscopic endometrial electrocision	HTF6U602	46.40	78	62	105	58560	5.75	0.00	2.61	276
	Hysteroscopic dissection of intrauterine adhesions	HTE7C601	43.50	78	62	131	58559	5.20	0.00	2.41	252
	Laparoscopic ovarian cystectomy	HTB6P501	40.70	69	53	105	58662	12.15	0.84	7.17	574
	Low-segment cesarean section	HUE6G303	36.40	59	48	130	59514	16.13	0.00	6.33	743
	Conization of uterine cervix	HTG6U401	36.00	67	47	53	57520	4.11	0.74	4.14	237
	Episiotomy	HTW7P701	20.00	40	22	34	59300	2.41	0.00	1.28	122
Ophthalmologic	Vitrectomy	HEQ6W301	48.60	79	71	288	67040	14.50	0.70	14.77	824
	Phacoemulsification and intraocular lens implantation	HEP6J302	43.20	79	63	203	66982	10.25	0.70	10.87	597
	Phacoemulsification	HEP6P301	37.80	69	55	123	66984	7.35	0.70	8.09	438
	Recession and resection of the rectus muscles	HEV7F301	35.50	64	55	192	67311	5.93	0.70	7.02	369
	Excision of eyelid mass	HED6U303	21.20	35	33	48	67800	1.41	0.80	1.49	87
Orthopedic surgery	Revision of total hip arthroplasty	HXD6L303	66.80	91	93	407	27132	25.69	0.69	19.03	1,215
	Total knee arthroplasty	HXJ6L304	58.00	86	83	407	27486	21.12	0.69	16.62	1,031
	Lumbar spinal canal decompression	HVT7M317	51.30	71	74	610	22558	23.53	0.69	15.89	1,062
	Anterior cervical corpectomy and bone graft fusion	HVH7M337	49.00	76	59	610	22554	17.69	0.69	14.61	887
	Total hip replacement	HXD6L301	48.00	81	54	326	27130	19.60	0.69	14.85	938
	Lumbar discectomy	HVU6U301	40.30	56	64	610	63030	12.00	0.76	11.75	690
	Arthroscopic meniscus repair	HXL7P502	36.70	71	26	326	29879	8.99	0.69	9.16	508
	Closed reduction of nasal bone fracture	HGB7H601	22.80	49	31	72	21337	3.39	0.69	5.15	248
Urological surgery	Laparoscopic radical prostatectomy	HSK6X501	62.70	89	84	500	55866	22.46	0.83	10.04	948
	Renal transplantation	HRB7T302	57.60	89	74	420	50365	46.13	0.83	29.32	2,235
	Laparoscopic partial nephrectomy	HRB6U501	52.30	83	66	350	50543	27.41	0.83	13.08	1,185
	Laparoscopic nephrectomy	HRB6W501	47.10	80	56	240	50545	25.06	0.83	11.10	1,055
	Laparoscopic partial adrenalectomy	HDF6U501	45.10	80	55	380	60650	20.73	0.84	10.35	918
	Transurethral ureteroscopic laser lithotripsy	HRF6P603	43.40	63	74	170	52353	7.50	0.00	3.03	348
	Transurethral electrocautery of bladder tumor	HRG7N401	27.80	47	40	150	52224	4.05	0.00	1.39	180
	Circumcision of prepuce	HSP6U301	24.40	35	38	30	54161	3.32	0.80	2.12	158
Otolaryngology	Cordectomy via endoscopic	HGP6U601	34.80	49	63	131	31545	6.30	0.00	3.52	325
	Transnasal endoscopic adenoidectomy	HGT6W601	32.20	49	59	62	42831	2.81	0.81	3.76	200
	Tonsillectomy	HGS6W401	22.10	35	38	60	42826	3.45	0.81	3.76	217

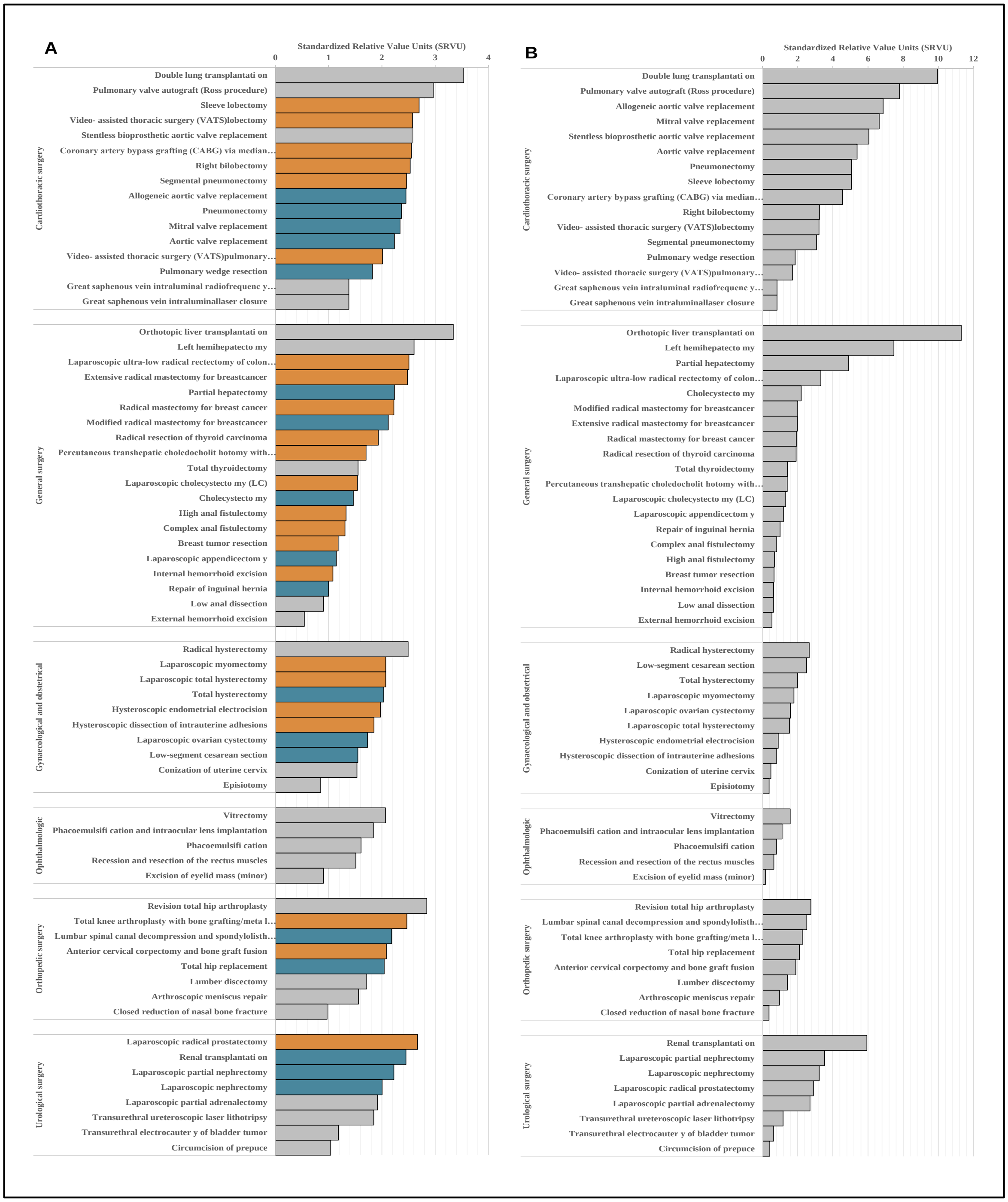

Supplementary Figure 1. The SRVUs across 6 Surgical Specialties for NHSIS and MPFS. **(A)** is the distribution of NHSIS-estimated SRVUs of surgical procedures across 6 specialties. **(B)** is the distribution of MPFS estimates for the same procedures. The gray bars indicate that the specialized value ranking of the surgery in NHSIS is consistent with MPFS. The range and blue bars indicate that the NHSIS ranks above and below the MPFS, respectively. SRVUs - standardized work relative value units. NHSIS - National Health Service Items Standard. MPFS - The U.S. Medicare Physician Fee Schedule.
